# The Impact of Chronic Inflammatory Enteropathy on Dogs’ Quality of Life and Dog-Owner Relationship

**DOI:** 10.3390/vetsci8080166

**Published:** 2021-08-19

**Authors:** Veronica Marchetti, Eleonora Gori, Valeria Mariotti, Angelo Gazzano, Chiara Mariti

**Affiliations:** Department of Veterinary Sciences, University of Pisa, 56124 Pisa, Italy; veronica.marchetti@unipi.it (V.M.); ele.gorivet@gmail.com (E.G.); valemar91@msn.com (V.M.); angelo.gazzano@unipi.it (A.G.)

**Keywords:** attachment, behavior, chronic enteropathy, dog, quality of life, relationship

## Abstract

This research was aimed at evaluating the impact of canine chronic enteropathies on dogs’ quality of life (QoL), their behavior, and owner–dog relationship. Forty-four dogs suffering from primary chronic enteropathies were assessed on the first visit with a veterinary gastroenterologist and on the first follow-up visit using a 1–10 visual scale to evaluate five features of QoL, the Canine Chronic Enteropathy Clinical Activity Index, the Lexington Attachment to Pet Scale, and the Canine Behavioral Assessment and Research Questionnaire. They were compared to a control group of 49 healthy dogs and to a group of 50 dogs suffering from cancer. QoL and severity of enteropathy were negatively associated; enteropathic dogs on the first visit had a lower QoL than healthy dogs for all features and a lower general QoL than cancer patients; enteropathic dogs on the follow-up visit improved significantly for general QoL, health QoL, and interaction QoL. Higher levels of attachment between the owner and the dog were obtained for dogs affected by chronic enteropathies. Finally, dogs showed higher scores for separation-related behaviors and contact/attention behaviors on the first visit than on the subsequent follow-up. As in human medicine, chronic enteropathies have a strong negative impact on dogs.

## 1. Introduction

Dogs have shared their life and story with human beings for several thousands of years [1] and today are considered, by most of their owners, family members. The process of domestication facilitated the development of a strong interspecific attachment bond, for many aspects mirroring the child–caregiver relationship [2,3]. Consequently, much more attention is paid to dog welfare, both from a physical and from a psychological point of view, by owners as well as by service providers (veterinary surgeons, nutritionists, etc.). In veterinary medicine, both a more specialistic knowledge and a more holistic and multidisciplinary approach to diseases are required, especially in canine gastroenterology, due to its connections with other veterinary branches and its relevance. In fact, a recent study reported that, in a very large sample of dogs attending veterinary clinics, gastrointestinal diseases were the most prevalent (17.8%), followed by dermatological and musculoskeletal diseases [4].

The term chronic inflammatory enteropathy is used for those subjects in which gastrointestinal symptoms (i.e., vomiting, diarrhea, borborygmi and flatulence, abdominal pain, and weight loss) have persisted for at least three weeks and are attributable to an intestinal inflammatory state [5]. Chronic enteropathies, retrospectively or based on the response to therapy, can be divided into specific categories: food-responsive, antibiotic-responsive, and immunosuppressant-responsive. In addition, a particular subgroup of enteropathies has to be considered, represented by a more serious condition characterized by an intestinal loss of proteins (protein-losing enteropathy), which in some cases may be responsive to diet but more frequently is responsive to immunosuppressive therapies. There are also cases that do not respond to any therapy and are indicated as affected by non-responsive enteropathy [6,7].

Chronic enteropathy, due to its prolonged and recurrent symptoms, might seriously compromise the quality of life of affected individual. In human medicine, Health-Related Quality of Life (HRQoL) is a broad concept that considers many aspects, such as patients’ physical health (also taking into account the pathology they are suffering from), their psychological condition, their ability to being independent from other people, and their social relationships [8]. HRQoL is considered an excellent indicator of the outcome of chronic diseases, including inflammatory bowel disease (IBD); for its correct evaluation, the therapy to which the patient is subjected has also to be considered [9].

The assessment of the Quality of Life (QoL) in the veterinary field is more difficult, as animals cannot report their perception of QoL [10]. The QoL assessment for pets is therefore carried out by the owners, based on the direct observation of their animal and on the interpretation of the pet behavior [11]. The assessment made by a proxy is quite similar to that in the pediatric field; the similarity is increased by the impact that the disease has not only on the subject, but also on the family where the patient lives [12].

The evaluation of QoL is based on positive and negative aspects of an animals’ life, that make it better or worse, referred to that specific animal [10]. HRQoL instead represents the quality of life with respect to a specific pathology the animal is suffering from [10]. The evaluation of both indexes is very important and can be relevant in the euthanasia choice process or in the evaluation of both therapy and necessity of further intervention [11].

Although QoL is sometimes considered a synonym for welfare and therefore includes the state of the animal (both physical and mental) and the possibility for the animal to display behaviors related to its nature and genetics [13], it is more correct to see the welfare state as a factor that can influence the QoL. In particular, a poor but short-term welfare state can impact the QoL to a slight extent, while a poor chronic welfare state can have a significant impact on the QoL [10]. This might also be true for chronic diseases: by reducing the welfare of the subject in the long term, chronic diseases could lead to a reduction in QoL. For instance, in human medicine, it has been shown that chronic intestinal pathologies can have a significant impact on HRQoL [9,13,14,15] as well as on behavioral aspects, with the possible onset of psychological–psychiatric disorders such as anxiety and depression [15,16,17]. As for dogs, specific studies on QoL are available mainly for oncological canine patients [18,19] and, in a retrospective study, for a population of enteropathic dogs [20]. 

The aim of this research was to evaluate the impact of canine primary chronic enteropathies on dogs’ quality of life, their behavior, and the owner–dog relationship.

## 2. Materials and Methods

This prospective research was carried out at the Veterinary Teaching Hospital “Mario Modenato” of the University of Pisa (Italy). A favorable opinion on this study was obtained from the Committee on Bioethics of the University of Pisa (Review No. 5/2021).

### 2.1. Participants

Three groups of animals, corresponding to an experimental group and two control groups, were involved in the research. 

The experimental group included dogs of both sexes, of any breed or mixed-breed, with a diagnosis of primary chronic enteropathy, i.e., dogs with gastrointestinal symptoms lasting at least three weeks, subjected to a path of exclusion of extraintestinal pathologies through a complete blood biochemical profile, coprological examination, and abdominal ultrasound [5]. All primary enteropathies such as food-responsive, antibiotic-responsive, immunosuppressant-responsive, and protein-losing enteropathies were therefore included. Dogs less than 11 months of age (for possible interference with the assessment of behavior), with acute enteropathies or chronic enteropathies secondary to other pathologies were excluded.

The experimental group of enteropathic dogs consisted of a total of 44 dogs, 20 females (9 neutered) and 24 males (1 neutered), 59.3 ± 45.0 months of age (mean ± standard deviation), of various breeds (German Shepherd, *n* = 8; Weimaraner, *n* = 4; Bolognese, French bulldog, Poodle, *n* = 3 for each breed; Golden retriever, *n* = 2; Great Dane, Basenji, Dachshund, Border Collie, Pug, Espagneul Breton, Cavalier King Charles Spaniel, Jack Russel terrier, Kurzhaar, Maltese, Rottweiler, English Setter, West Highland White Terrier, *n* = 1 for each breed) or mixed-breed (*n* = 7).

The first control group consisted of 50 dogs affected by an oncological disease, 29 females (18 castrated) and 21 males (6 castrated), 125.2 ± 35.6 months of age, of various breeds (German Shepherd, *n* = 3; Beagle, Labrador, Schnauzer, Italian Hound, English Setter, *n* = 2 for each breed, Great Dane, American Staffordshire terrier, Poodle, Bernese Mountain dog, Boxer, Cocker Spaniel, Dobermann Pinscher, Dogo Argentino, Golden Retriever, Jack Russel Terrier, Lagotto Romagnolo, Maremma Shepherd, Pinscher, Pit Bull, Rottweiler, Shar Pei, Terranova; *n* = 1 for each breed) or mixed breed (*n* = 18). The oncologic diagnosis was lymphoma (*n* = 13), hemangiosarcoma (*n* = 7), mast cell tumor (*n* = 7), soft tissue sarcoma (*n* = 5), pulmonary adenocarcinoma (*n* = 3), osteosarcoma (*n* = 3), mammary carcinoma (*n* = 3), squamous cell carcinoma (*n* = 2), hepatic carcinoma (*n* = 1), gastric carcinoma (*n* = 1), anal sac carcinoma (*n* = 1), metastatic chondrosarcoma (*n* = 1), and hemangiopericytoma (*n* = 1). They were recruited during their first visit with a veterinary oncologist at the same Veterinary Teaching Hospital.

The second control group consisted of 49 dogs that owners indicated to be healthy through items in a questionnaire specifically asking if the dog was healthy and investigating the presence of signs of acute/chronic diseases (based on such items, 4 dogs from the original group of 53 were excluded). This group was composed of 33 females (19 neutered) and 16 males (4 neutered), 64.0 ± 44.9 months of age, of various breeds, i.e., Jack Russell Terrier, *n* = 4; Griffone Korthals, Dachshund, Border Collie, *n* = 3 each breed; Labrador retriever, Maremma Shepherd, Shiba Inu, Pinscher, Minitaure Schnauzer *n* = 2 for each breed; Poodle, Chihuahua, Golden retriever, Irish soft coated Wheaten Terrier, Belgian Shepherd, Australian Shepherd, German Shepherd, Spitz, *n* = 1 for each breed) or mixed-breeds (*n* = 18). The owners of this control group were mainly recruited among faculty members and students of the Dept. Of Veterinary Sciences, University of Pisa, the rest being personal contacts of the authors; this kind of recruitment was chosen in order to increase the likelihood of having dogs regularly checked by a veterinarian and, therefore really healthy, albeit the lack of a concurrent clinical exam. Only for the purpose of this article, these dogs will be referred to as healthy.

### 2.2. Assessment Tools

**Figure 1:**
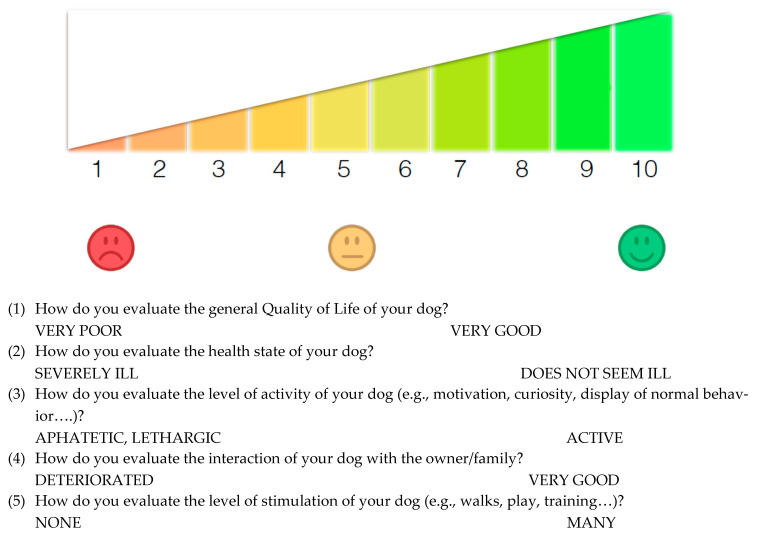
The 1–10 visual scale used to evaluate dogs’ QoL. For each of the five investigated features, the item was followed by the figure reported below and by a description of the scale extremes.

The assessment of the behavioral, relational, and quality of life aspects was carried out based on the answers obtained from three questionnaires:

- Canine Behavioral Assessment and Research Questionnaire (C-BARQ, https://vetapps.vet.upenn.edu/cbarq/; Accessed on 10 June 2021), in particular, its reduced form composed of 42 items, validated by Duffy et al. [21]. The C-Barq42 was completed for enteropathic dogs only, at the time of the first visit with a veterinary gastroenterologist at the Veterinary Teaching Hospital “M. Modenato”, University of Pisa (Italy), and again on the first follow-up visit, after about one month
- Lexington Attachment to Pets Scale (LAPS) [22]. It was completed by the owners of the healthy subjects and of the experimental group, the latter on the first visit and again at follow-up
- Visual Scale for the evaluation of the QoL, on a 1–10 scale (see Figure 1), divided into five questions concerning different aspects of QoL. The scale was created purposely for the current study and proved good reliability (Cronbach’s alpha = 0.87) and a non-normal distribution (skewness = −1.42; kurtosis = 1.73). It was completed by the owners of the experimental group on the first visit and on the follow-up, as well as by the owners of the healthy subjects and, for oncological patients, on the first visit with a veterinary oncologist at the same veterinary hospital.

For each enteropathic canine patient, the severity of enteropathy was determined twice, on the first visit and at follow-up, using the CCECAI (Canine Chronic Enteropathy Clinical Activity Index) [23]. The CCECAI scoring system assessed 9 categories of disease severity, including attitude and activity (from 0 [normal] to 3 [severely decreased]), appetite (from 0 [normal] to 3 [severely decreased]), vomiting (from 0 [<1 event/week] to 3 [>3 events/week]), fecal consistency (from 0 [normal] to 3 [watery diarrhea]), fecal frequency (from 0 [normal; <2 events/day] to 3 [severely increased; >5 events/day]), weight loss (from 0 [none] to 3 [>10%]), serum albumin concentration (from 0 [>2 g/dL] to 3 [<1.2 g/dL]), peripheral edema and ascites (from 0 [none] to 3 [severe]), and pruritus (from 0 [none] to 3 [pruritus regularly wakes dog]). Based on the CCECAI value, the severity was assessed as insignificant (0–3), mild (4–5), moderate (6–8), severe (9–11), and very severe disease (>12) [23]. In this study, we decided to group the 5 categories mentioned above into two categories: clinically insignificant–mild disease (CCECAI 0–5) and moderate-to-very severe disease (CCECAI ≥ 6).

The response to therapies was evaluated by calculating ΔCCECAI% (difference between the CCECAI on the first visit and that at the follow-up, divided by the value of the CCECAI on the first visit and then multiplied by 100) and defining it complete response when ΔCCECAI > 75%, partial response when ΔCCECAI was between 25 and 75%, and no response when ΔCCECAI < 25% [24]. Partial and complete responders were assigned to the responders group.

### 2.3. Statistical Analysis

The three groups were compared for age and weight using the Kruskal–Wallis test, with pairwise comparisons with the Bonferroni correction test.

Supposing that, not only in oncological dogs, but also in enteropathic dogs the disease might have a significant impact on QoL, differences in QoL between the different study groups (healthy, oncological, and enteropathic) were assessed using the Kruskal–Wallis test with pairwise comparisons with the Bonferroni correction test.

We hypothesized that the presence of a chronic enteropathy might affect the relationship between owner and dog; thus, the LAPS score was compared between healthy dogs and enteropathic dogs using the Mann–Whitney U-test.

In addition, for the experimental group, we hypothesized that dogs with higher disease severity (high CCECAI) might have significantly different LAPS, C-BARQ, and QoL scores compared to dogs with milder diseases. For this reason, considering the CCECAI severity categories, the Mann–Whitney U-test was used to evaluate differences between LAPS, C-BARQ, and QoL scores in dogs with clinically insignificant–mild disease (CCECAI 0–5) and moderate-to-very severe disease (CCECAI ≥ 6). The Wilcoxon rank test was used to compare enteropathic dogs’ CCECAI, LAPS, C-BARQ, and QoL scores of the first visit versus those of the follow-up. Lastly, the LAPS, C-BARQ, and QoL scores were also compared between responder and non-responder enteropathic dogs using the Mann–Whitney U-test. A *p*-value < 0.05 was considered statistically significant.

## 3. Results

There were no significant differences in age (*p* = 0.520) and weight (*p* = 0.970) between enteropathic and healthy dogs. Oncologic dogs resulted older and with a higher weight compared to both enteropathic dogs (*p* < 0.001 and *p* = 0.008, respectively) and healthy dogs (*p* < 0.001 and *p* = 0.004, respectively). 

**Figure 2:**
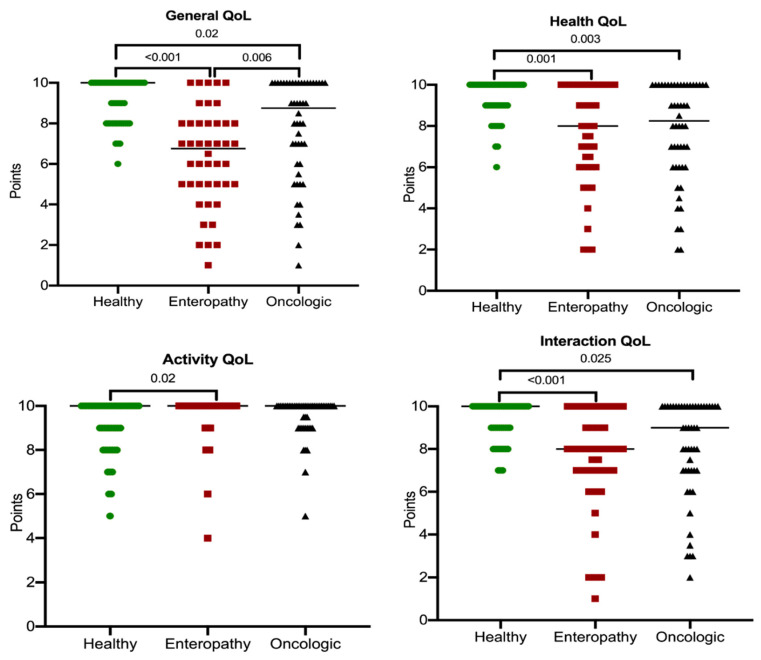
Statistically significant QoL scores (general, health, activity, and interaction) for healthy, enteropathic, and oncologic dogs.

Comparing the three groups of dogs (Figure 2), a statistically significant difference emerged in terms of general QoL (*p* < 0.001), health QoL (*p* < 0.001), activity QoL (*p* = 0.022), and QoL interaction (*p* < 0.001). Stimulation QoL resulted to be not significantly different among the three groups (*p* = 0.400).

In the enteropathic group, the severity of the disease was assessed on the first visit and at the subsequent follow-up using the CCECAI clinical score. The median CCECAI on the first visit was 4.5 and, on the control visit, it was 1. The CCECAI values on the follow-up were statistically lower than those determined on the first visit (*p* < 0.001), and a clinical response was achieved in 75% of the cases.

Regarding the QoL of enteropathic dogs, the scores of three out of five items were significantly higher on the follow-up compared to the first visit (Table 1).

The severity of the enteropathy, as evaluated by CCECAI values obtained on the first visit, was negatively associated with QoL: dogs with a clinically insignificant–mild illness dogs had a statistically higher QoL than moderate-to-very severely ill dogs, for all the five investigated features (Table 2).

For the Lexington Attachment to Pets Scale (LAPS), the median score obtained in enteropathic subjects on the first visit was 61.5 (range 25–69) and in healthy subjects it was 58 (range 39–69), with a statistically significant difference (*p* = 0.030). On the other hand, there was no statistically significant difference for LAPS in enteropathic subjects between the first visit and the follow-up (*p* = 0.15). Regarding the comparison between responders and non-responders on the follow-up visit, no significant differences in the LAPS score between non-responders and responders was found.

In enteropathic dogs, the scores of C-BARQ for behaviors related to separation (*p* = 0.024) and attention/contact seeking (*p* = 0.002) were higher on the first visit than on the follow-up. No significant differences emerged between the scores of the remaining behavioral categories of the C-BARQ and between responders and non-responders.

## 4. Discussion

The data of the current study suggest that QoL, dog behavior, and dog–owner relationship are affected by the presence of a chronic enteropathy in dogs.

Concerning the QoL, first of all, it was found that dogs with a moderate-to-very severe disease had lower QoL scores than dogs with an insignificant–mild disease, suggesting that the severity of the enteropathy is negatively associated with the QoL of the patient: the severer the enteropathy, the lower the dog’s QoL. These data may indicate that, as in humans with inflammatory bowel disease [9], chronic enteropathies can also have a significant impact on the QoL of dogs, due to chronic symptoms and the need of long therapies and periodic follow-ups, the latter being stressful themselves [25,26]. It is interesting to note that, at least in humans, due to the so-called gut–brain axis, intestinal diseases can have serious repercussions on the patient, including the development of psychological disorders such as anxiety and depression; these can in turn affect intestinal functionality [16,27]. It is desirable that future research focuses on these aspects to better understand the overall implications of chronic enteropathies in dogs.

Secondly, it was found that enteropathic dogs on the first visit had a lower QoL than healthy dogs for all variables and a lower QoL than the general QoL of oncologic patients. The first result is certainly very important and scarcely reported in the scientific literature, despite the impact gastrointestinal problems on animals’ QoL. The comparison with oncologic patients, on the other hand, provided rather unexpected results. In fact, the control group of oncological dogs was recruited as the impact of oncological disease on patients’ QoL, including canine patients, is well-recognized [18,19].

However, the current study suggests that, despite the diagnosis of an oncologic disease having a significant emotional impact on dogs’ owners, the affected dogs had a better general QoL than patients with chronic gastrointestinal disorders (as long as the disease allowed it). Due to the impact of cancer on QoL, the lack of differences found between oncologic dogs and enteropathic ones for items relating to QoL other than general QoL confirms to which extent chronic enteropathies have an effect on the various aspects that characterize the quality of life of a dog. It must be underlined that, in this study, the QoL was evaluated in oncological patients on the first visit; such diagnosis prompts treatment and raises owners’ attention to the problem, when the disease is not hardly impacting on the dog, as it is in more advanced phases. On the contrary, some owners of dogs suffering from enteropathy might neglect or underestimate the symptoms, at least until problems arise in the management of the dog and therefore create a direct disturbance to the owners themselves. In support to this information, Craven et al. [20] reported the presence of gastrointestinal symptoms as early as 9.5 years before diagnosis. This clearly allows for an aggravation of the disease and consequently a reduction in QoL, which could be prevented by an early action of the owners and an early detection of a reduced QoL.

Oncologic dogs were reported to have a lower QoL than healthy dogs, and such data agree with previous literature on the topic [18,19]. The assessment of QoL in the oncologic patient is important to decide and modulate therapeutic choices [28] as well as to understand the effects of disease and treatment on the animal [29]. It is possible that the same approach used for enteropathic patients could lead to an improvement in therapy and QoL of the dog.

Thirdly, comparing the scores of the five QoL indexes obtained for the same subjects on the first visit and at the follow-up, the results improved significantly for general QoL, health QoL, and interaction QoL. In contrast, no significant differences emerged between the scores obtained for QoL activity and QoL stimulation. This is probably due to the rather high QoL score for activity and stimulation (greater than 8 out of 10) that dogs presented on the first visit, despite their pathology, leaving less room for an improvement.

In the only veterinary study on QoL in enteropathic dogs (to the authors’ knowledge), QoL assessment was carried out through a telephone questionnaire in which the owner was asked to rate the dog’s QoL on a scale from 1 to 10 at the time of the call and, retrospectively, at the time of diagnosis [20]. In that study by Craven et al. [20], an improvement in QoL at follow-up was also observed in most of the 80 dogs involved, with the exception of 5% of cases where it remained unchanged.

As for the severity of the enteropathy, our data showed a decrease in the CCECAI score at the follow-up compared to the first visit, indicative of an improvement in the animal response to the administered therapy; in 55% of cases, a complete response to therapy was observed. This improvement indicates that, once the right therapeutic treatment has started, most of the subjects already have a good response at the subsequent follow-up, with important repercussions on their QoL.

The current study also evaluated the possible impact of chronic enteropathies on the dog–owner relationship. Higher scores for the LAPS [22], indicating a higher level of attachment of the owner to the dog, were obtained for dogs affected by chronic enteropathies compared to healthy dogs. No difference was observed when comparing the LAPS scores of the whole group of enteropathic dogs on the first visit and at follow-up or between responders and non-responders. The higher scores obtained for enteropathic dogs was likely due to the disease itself, as higher scores of LAPS have been found to be associated with owners’ willingness to ensure better care for their dogs [30].

Finally, concerning dog behavior, C-BARQ comparison between the first visit and the follow-up visit showed higher scores for separation-related behaviors and contact/attention behaviors in the former. These results are in agreement with the secure-base role that owners can play for their dogs [2] and the complex bond dogs establish with their owners [31], thus increasing the search for proximity in the times the dog feels ill. In addition, dogs with a chronic enteropathy might increase the number of times they evacuate and therefore they might increase the attention paid to the owners to satisfy their need.

The results of the current study basically confirm what could be hypothesized through a parallelism with human chronic enteropathies and are in agreement with the scarce scientific literature available on dogs. However, the relatively small sample and the potential interference of factors not investigated in the current study (especially, dog and owner details, such as age, gender, length of their relationship, etc.) call for caution as well as for more studies to improve our knowledge about chronic enteropathies and QoL in dogs. Another limitation of this study is the incomplete assessment of the health status of the control group indicated as healthy; although some measures were taken by authors to reduce the likelihood of including dogs that were not completely healthy, a more rigorous check would make the comparison with this group more robust.

## 5. Conclusions

For the first time in veterinary medicine, the quality of life of dogs with chronic enteropathy was evaluated using specific questionnaires. The results of the current study suggest that QoL, dog behavior, and dog–owner relationship are all affected by the presence of a chronic enteropathy in dogs.

In detail, dogs’ QoL is negatively influenced by the presence of a chronic enteropathy and by its severity, and dogs suffering from a chronic enteropathy have a lower QoL than healthy dogs, and, at least for its general feature, than dogs with an oncological disease. The bond between owner and dog results in owners being more attached to enteropathic dogs than to healthy ones. The dog–owner relationship might be affected as well, as the presence of a chronic intestinal disease seems to modify dogs’ behavior, so that dogs seek the owner more often; however, these results can be read using different keys, such as the theory of attachment or the fact that the dog simply needs to go out more frequently, one not excluding the other. The increased demand for attention and separation-related behaviors in canine enteropathic patients deserves further investigation, also considering the frequency of gastrointestinal disorders in dogs with separation-related problems.

The results of this study provide a modest but useful piece of information for veterinarians in practice.

## Figures and Tables

**Table 1:** Comparison of QoL scores on first visit and follow-up in enteropathic dogs (* = *p* < 0.05).

	First Visit			Follow–Up			
	Mean	Median	Range	Mean	Median	Range	*p*-Value
General QoL	6.33	6.75	1–10	7.27	8	2–10	0.004 *
Health QoL	7.52	8	2–10	8.33	9	2–10	0.044 *
Activity QoL	9.55	10	4–10	9.32	10	4–10	0.165
Interaction QoL	7.57	7.77	1–10	8.2	9	2–10	0.043 *
Stimulation QoL	8.23	9	2–10	8.53	9	3–10	0.585

**Table 2:** Results of QoL scores on the first visit in the enteropathic group based on CCECAI groups (0–5 and ≥ 6) (* = *p* < 0.05).

	CCECAI 0–5	CCECAI ≥ 6	*p*-Value
General QoL	7 (4–10)	5 (1–8)	0.001 *
Health QoL	9 (4–10)	6 (2–10)	0.003 *
Activity QoL	10 (8–10)	10 (4–10)	0.004 *
Interaction QoL	8.5 (6–10)	7 (1–10)	0.001 *
Stimulation QoL	9.5 (6–10)	7.5 (2–10)	0.002 *

## Data Availability

Data are available on request.
